# From empathy to action: exploring emotional mechanisms of online public opinion in a public health crisis

**DOI:** 10.3389/fpubh.2025.1721245

**Published:** 2026-01-05

**Authors:** Adina Yuetikuer

**Affiliations:** School of Journalism, Fudan University, Shanghai, China

**Keywords:** digital empathy, emotional valence, online behavior, public health crisis, sentiment analysis, Weibo

## Abstract

**Introduction:**

Drawing on digital empathy theory, this study explores how empathic expressions evolve and manifest as action-oriented behaviors in online public discourse during a public health crisis. Specifically, it focuses on a doctor’s suicide incident discussed on the Weibo platform, examining how various empathy types influence public opinion and engagement. By investigating the emotional and behavioral responses within the digital realm, the study contributes to understanding the complex dynamics of empathy and its impact on collective emotion and decision-making during health-related crises.

**Methods:**

Data were collected from Weibo posts related to the doctor’s suicide incident, and analyzed using Latent Dirichlet Allocation (LDA) topic modeling and sentiment analysis.

**Results:**

The study revealed four primary areas of focus in the posts: medical decision-making disputes, cyberbullying and platform responsibility, media reproduction, and official reports and judicial investigations. Additionally, the study identified four distinct types of empathy expressed in the comments: professional empathy, institutional empathy, cultural empathy, and action-oriented empathy. The study also revealed significant differences in emotional valence across the four empathy types, and found that female users exhibited a higher level of empathic engagement compared to male users.

**Discussion:**

These findings suggest that digital empathy in public health crises operates as a complex, multi-layered emotional-behavioral mechanism. Different empathy types not only influence the emotional tone of online discussions but also affect the level of engagement and the type of actions that users take. This study highlights the critical role of empathy in shaping online behaviors during health-related crises and provides valuable insights into how platforms can manage emotional engagement to encourage constructive participation.

## 1 Introduction

In recent years, public health incidents have repeatedly triggered intense online discussions in China, where medical risks, information asymmetry, and digital amplification shape the doctor–patient relationship. With the rise of platforms such as Weibo and TikTok, public discourse around medical controversies has become increasingly emotionalized, visualized, and accelerated through algorithmically mediated interactions. Within these environments, empathy emerges as a central mechanism driving how users perceive events, express emotions, and participate in collective discussions.

The “Zhoukou doctor suicide incident” exemplifies this dynamic. After saving a patient experiencing amniotic fluid embolism, the doctor faced persistent online harassment from the patient’s family and eventually took her own life. The incident quickly went viral following official statements and media coverage, sparking widespread debate over medical decision-making, cyberbullying, and institutional responsibility. Unlike earlier one-dimensional online outrage, this event revealed diverse forms of empathic responses, ranging from professional support for the doctor to moral judgment and calls for action.

Digital empathy offers a valuable framework for understanding how emotions unfold and take shape in such online environments. Although previous research has explored digital empathy in contexts such as health communication and online support, its thematic patterns, emotional tendencies, and behavioral manifestations during public health crises remain underexplored.

To address this gap, this study examines the Zhoukou incident as a case of online public opinion formation. By analyzing Weibo discussions, it aims to answer three questions:

*RQ1*. What types of digital empathy are manifested in public discussions surrounding the incident?

*RQ2*. How do different media frames and emotional expressions influence user engagement in discussions of this public health crisis?

*RQ3*. In what ways does digital empathy evolve from emotional resonance to collective actions in the process of opinion formation?

## 2 Literature review

### 2.1 The concept and evolution of digital empathy

Empathy originated in the fields of psychology and social neuroscience and is regarded as a fundamental psychological mechanism underlying human sociality and prosocial behavior. It is commonly defined as the process by which individuals perceive, understand, and emotionally resonate with the emotional states of others, thereby motivating understanding, compassion, and helping behaviors (1, 2).

Classic theories conceptualize empathy as comprising two primary dimensions: emotional empathy and cognitive empathy. *Emotional empathy* emphasizes affective resonance and the capacity to vicariously experience others’ emotions such as sympathy, compassion, or anger (3, 4). *Cognitive empathy*, in contrast, focuses on perspective-taking and role-shifting at the rational level, reflecting the ability to comprehend others’ situations, intentions, and motivations (5). The interplay between these two dimensions forms the psychological foundation for social interaction, moral judgment, and prosocial decision-making (6).

With the digitalization of the communication environment and the proliferation of social media, scholars have increasingly focused on the transformation and extension of empathy within online contexts, leading to the emergence of the concept of digital empathy. Digital empathy is defined as the ability of individuals to perceive, understand, and respond to others’ emotions in social media, online communities, and virtual interaction environments through textual messages, emojis, images, videos, and algorithmic recommendation mechanisms (7, 8).

This form of empathy is not merely an affective experience at the psychological level but also a form of mediated interaction and social practice, encompassing multiple dimensions such as informational expression, symbolic interaction, and social connection (9).

Compared with traditional face-to-face empathy, digital empathy exhibits three defining characteristics.

First, mediated expression, whereby emotions are conveyed and perceived through technological interfaces and symbolic cues (e.g., text, emojis, and videos), with the process being shaped by interface design and platform mechanisms (10).

Second, decontextualized interaction, as digital spaces often lack nonverbal cues and contextual information, leading to potential ambiguities and misinterpretations in empathic communication (11).

Third, visibility amplification, where platform algorithms tend to prioritize and amplify emotionally charged content, transforming emotional expressions into quantifiable interaction data—such as likes, shares, and comments—that serve as indicators of social status and group affiliation (12, 13).

Therefore, digital empathy should be understood not only as an internal emotional experience but also as a socially constructed practice shaped by algorithmic and interactional logics. It is simultaneously influenced by technological mediation and platform governance, while exerting influence on emotional expression, group identity, and public discourse formation (43). Within digital public spheres, empathy is transformed into visible interactive behaviors and social signals, becoming a critical driving force for emotional aggregation and collective action.

### 2.2 The structure and typology of digital empathy

Scholars generally agree that digital empathy is a multidimensional construct that integrates cognitive, affective, and behavioral components. The mainstream perspective conceptualizes digital empathy as comprising cognitive empathy, emotional empathy, and behavioral or active empathy (14, 15).

Cognitive empathy refers to an individual’s rational understanding and perspective-taking toward others’ situations. It involves analyzing contextual information, adopting others’ viewpoints, and using logical reasoning to infer the emotional states and motivations of others (5).

Emotional empathy, emphasizes affective resonance with others’ emotional experiences—such as compassion, sympathy, or anger—and constitutes the emotional foundation for resonance and diffusion on social media platforms (3, 9).

Behavioral or active empathy highlights the translation of internal emotional experiences into observable social actions. Through behaviors such as posting comforting messages, liking, sharing, or participating in online advocacy, individuals externalize their empathy, turning emotional resonance into visible practices (10).

In the context of social media, behavioral empathy becomes particularly salient: users often employ symbolic interactions (e.g., hashtags, emojis, or supportive comments) to express their emotional stance and foster emotional visibility and social connectedness. This process transforms empathy from an internal psychological response into a public, performative, and participatory practice shaped by platform affordances.

In recent years, scholars have further introduced new conceptualizations such as structural empathy and algorithmic empathy to reveal how digital empathy evolves under the influence of platform architectures and algorithmic logics. Structural empathy emphasizes that emotional expression and perception are no longer solely driven by individual psychological processes but are systematically shaped by platform infrastructures, interface design, and information flow mechanisms (16). Algorithmic empathy, on the other hand, refers to the ways in which platforms amplify particular emotional content through recommendation systems and affect-oriented prioritization mechanisms, fostering algorithmic resonance that shapes users’ emotional biases and collective sentiments [Lupton, 2021; (13)]. These perspectives highlight the sociotechnical coupling of digital empathy, underscoring how technological mediation exerts structural influences on emotional expression and public opinion formation.

Across different domains, digital empathy demonstrates diverse manifestations.

In educational technology, empathic interaction design has been found to enhance learning motivation, foster social connectedness, and facilitate emotional resonance and collaboration among learners (15, 17).

In the mental health field, online empathic interactions have been shown to alleviate loneliness, anxiety, and depression by building supportive psychological networks—effects that became particularly salient during crisis contexts such as the COVID-19 pandemic (18).

In media and communication studies, digital empathy is regarded as a key driver of public engagement and issue diffusion, promoting emotional resonance, stimulating collective action, and strengthening group identity (19).

Overall, digital empathy can be conceptualized as a dynamic composite integrating cognition, emotion, and behavior. It embodies both internal emotional experiences and external social practices, shaped simultaneously by individual psychological mechanisms and platform technological logics.

### 2.3 Emotional diffusion and algorithmic mechanisms on social media

In social media environments, emotions function not only as individual psychological responses but also as key drivers of information diffusion. Prior studies have shown that high-arousal emotional content attracts greater attention and spreads more widely than neutral information (12, 20). Emotions such as anger, sadness, and sympathy often serve as triggers for public opinion formation, rapidly aggregating attention and participation. The advantage of emotional content, when combined with the interactive features of social media, leads to an increasing emotionalization of public expression, which may further push online discourse toward polarization or empathic convergence.

At the platform level, recommendation algorithms prioritize emotionally charged content by weighting engagement metrics such as likes, comments, and shares, resulting in a phenomenon often referred to as the “emotional amplification loop” (21). This process implies that emotional expressions are no longer purely organic but are integrated into the logic of algorithmic recommendation, becoming critical determinants of content visibility and reach. In other words, platforms transform emotions into computable and visualized signals through datafication, imbuing them with structural significance in the dynamics of social diffusion (16).

Empirical research further shows that emotional content—especially moral outrage—spreads more rapidly and broadly in online networks, reinforcing algorithmic amplification dynamics (22). Similarly, large-scale analyses of Twitter demonstrate that emotionally salient misinformation diffuses faster and deeper than factual content, partly due to algorithmic reinforcement of high-engagement posts (23). Together, these findings illustrate a bidirectional process in which algorithms shape emotional visibility, while collective emotional expressions continuously feed back into algorithmic ranking systems, forming a mutually reinforcing loop.

This algorithm–emotion coupling mechanism endows empathic expression with the characteristics of strategic action (13). Users are increasingly aware that their emotional expressions and interactive behaviors can influence algorithmic recommendations, thereby indirectly shaping event salience and the structure of public opinion. The emerging theory of algorithmic awareness further elucidates this phenomenon. Research suggests that users are consciously aware of the selective distribution of algorithms and often adopt algorithmically favorable expression strategies to enhance the visibility and reach of their content (24).

In practical terms, this awareness motivates users to integrate empathic expression with behavioral mobilization. During public debates or crisis events, users frequently engage in actions such as liking, commenting “push this up,” and sharing posts, transforming emotional resonance into action-oriented empathy aimed at boosting algorithmic weight and enhancing event visibility (25, 26). This demonstrates that digital empathy is no longer merely an internal emotional reaction; rather, it is embedded within algorithmically driven social ecosystems, manifesting as a form of both authentic and strategic social action.

Overall, emotional diffusion and algorithmic mechanisms form a dual driving force in social media environments. On the one hand, emotions serve as central resources that accelerate the aggregation and dissemination of public sentiment; on the other hand, platform algorithms convert empathic expressions into explicit forces of opinion construction through datafication and recommendation logic. This process underscores the socio-technical coupling of digital empathy, wherein emotions function simultaneously as natural psychological responses and strategic behaviors shaped by algorithmic logic.

### 2.4 Digital empathy in public health communication and online discourse

In public health crises, social media has become a central arena for emotional expression, information exchange, and public opinion formation (27). When confronted with health risks and emergencies, individuals tend to engage in online interactions that foster emotional resonance and collective identity (28). Studies have shown that during major public health emergencies such as the COVID-19 pandemic, digital empathy was widely manifested on social media platforms. Users shared personal prevention experiences, expressed condolences and encouragement, and reposted authoritative information, thereby forming virtual support networks based on emotional connection (29). These empathic interactions not only expanded the breadth and depth of information dissemination but also enhanced collective risk perception and social cohesion (30).

Moreover, digital empathy within public health discourse exhibits issue sensitivity and a multi-layered structure. In the context of social conflicts and value-laden controversies, public empathic expressions often blend understanding, anger, and institutional critique. For instance, in medical disputes or vaccine controversies, users may simultaneously express sympathy for the individuals involved and raise concerns about fairness, transparency, and accountability in healthcare systems (31, 32). This pattern suggests that digital empathy functions not only as an emotional reaction but also as an affective response to institutional trust and social justice.

Furthermore, gender differences are salient in empathic expression. Research indicates that female users are more inclined to use emotionally expressive language, demonstrating care, support, and higher levels of interactive engagement. In contrast, male users tend to display cognitive empathy, focusing on rational analysis and critical discussion (33, 34). These findings highlight the diversity of digital empathy practices across demographic groups and underscore the importance of social and cultural factors in shaping emotional expression during public health crises.

Media and institutional empathic reporting also play a crucial role in shaping public health discourse. Empathic journalism, through storytelling and emotional narratives, can enhance public understanding and emotional resonance, alleviate panic and uncertainty, and foster institutional trust during crises (35). However, algorithmic recommendation systems may unintentionally reinforce homogeneous perspectives and emotional biases, leading individuals to be increasingly exposed to content aligned with their preexisting views. This process contributes to public opinion polarization and affective fragmentation (36).

Overall, digital empathy in public health communication demonstrates a multi-layered structure, evolving from individual emotional resonance to institutional critique, and further to action-oriented expressions. It reflects not only the emotional responses of the public to health risks and social issues but also the ways in which individuals participate in opinion formation through algorithmic awareness and interactive behaviors in digital environments. Nevertheless, existing studies predominantly focus on single-dimensional empathic functions, lacking systematic empirical analyses of its generation mechanisms, emotional pathways, and algorithmic interactions.

Despite the growing body of research on digital empathy, several limitations remain. First, prior studies often isolate functional aspects of empathy—such as emotional support or empathic journalism—without explaining how empathy is activated, shaped, or transformed within algorithm-driven social media environments. Second, existing work largely centers on institutional or media perspectives, overlooking how ordinary users articulate and enact empathy in public discussions. Third, few studies have examined how different forms of empathy—cognitive, emotional, and behavioral—coexist and interact in public health controversies, where emotional resonance frequently intersects with anger, distrust, and calls for accountability.

To address these gaps, this study draws on the Zhoukou doctor suicide incident to investigate the thematic structure, emotional tendencies, and empathic expressions in online public discourse. Based on literature on emotional diffusion and digital empathy, the following hypotheses are proposed:

*H1*. Different themes in public discussions will exhibit distinct emotional profiles.

*H2*. Emotional expressions will significantly differ across types of digital empathy.

*H3*. Female and male users will differ in their expressions of digital empathy.

These hypotheses aim to clarify the structural features, emotional pathways, and gendered patterns of digital empathy within the evolving dynamics of online public opinion.

## 3 Materials and methods

This study takes the “Zhoukou doctor suicide incident” as a case to examine the dynamics of online public opinion and empathic expressions in a public health crisis. A pregnant woman at Zhoukou Sixth People’s Hospital in Henan Province suffered from amniotic fluid embolism during delivery. To save her life, the attending doctor performed a hysterectomy. Although the patient survived, her family expressed dissatisfaction, claiming the doctor had not obtained full consent and accusing the hospital of depriving the woman of her ability to bear more children. Since late 2024, the family had intermittently posted short videos on platforms such as Douyin, framing the case as a “medical dispute.” These early videos, however, attracted limited attention and did not trigger large-scale public discussion.

On the evening of August 1, 2025, the doctor, Ms. Shao, tragically took her own life by jumping from the hospital building. On August 5, the local Health Commission issued an official statement confirming that the doctor had been subjected to long-term cyberbullying, ruling out medical malpractice, and launching a formal investigation. Following extensive media coverage and authoritative responses, the event quickly went viral on Weibo, topping the trending list. Mainstream outlets called for strict punishment of cyberbullying and legal protection for healthcare professionals, further amplifying public attention and transforming the case into a nationwide topic.

Although early content appeared on short-video platforms, Weibo was chosen as the primary data source. Weibo provides the most open and traceable text-based discussion environment among Chinese social media platforms. Public crises in China often trigger large-scale debate on Weibo, where users post comments, emotional narratives, and opinion-driven discussions. These features make Weibo more suitable for topic modeling analysis than video-centered platforms, which rely heavily on audiovisual content and fragmented comment streams.

To build the dataset, the researchers first browsed early discussions on Weibo to identify commonly used hashtags and search terms. After reviewing a substantial number of posts, it was found that event-related discussions repeatedly used a stable set of hashtags, which allowed us to capture the full scope of the conversation. Based on this preliminary exploration, relevant posts were extracted using core keywords such as “Zhoukou doctor suicide,” “cyberbullying against doctor,” and “Zhoukou Sixth Hospital childbirth,” together with the main event hashtags (e.g., #Official Report On Zhoukou Doctor Suicide#, #Doctor Committed Suicide After Saving Patient With Amniotic Fluid Embolism#). The dataset covers posts and comments published between August 5 and August 31, 2025, including media reports, institutional statements, and user-generated content. After manual review and cleaning (removing advertisements, duplicates, irrelevant or invalid entries), the final corpus contains 1,877 Weibo posts and 16,428 comments.

For analysis, the study employed Jieba for Chinese word segmentation and stop-word removal. Latent Dirichlet Allocation (LDA), a probabilistic topic modeling technique that identifies clusters of co-occurring words to reveal latent thematic structures within large text corpora, was applied to extract major discussion topics from posts and comments. While LDA efficiently uncovers semantic patterns in unstructured text, it assumes topic independence and may overlook contextual nuances or implicit meanings in social media discourse. Specifically, LDA hyperparameters followed empirical standards: *α* = 50/K, *β* = 0.01, with 1,000 iterations for convergence. Optimal K for posts and comments was determined via perplexity and topic coherence, referenced to corresponding curves.

For sentiment analysis, this study used two widely applied Chinese sentiment lexicons: the HowNet sentiment lexicon and the Tsinghua University sentiment lexicon. These lexicons have been broadly adopted in Chinese text-mining research and provide stable coverage for online public-opinion analysis. Following previous work that applied lexicon-based methods in large-scale social-media studies (37), we categorized each post and comment into positive, neutral, or negative sentiment based on keyword matching after segmentation and cleaning. While efficient for large-scale text analysis, this approach may overlook subtle or culturally specific emotional cues often present in online discourse. However, lexicon-based sentiment analysis remains a widely accepted method for Chinese-language research due to its transparency, scalability, and linguistic adaptability.

To ensure analytical reliability, two researchers independently validated the LDA-derived topics and sentiment classifications, indicating satisfactory classification accuracy. The mapping from comment topics to empathy types was then refined through team discussion, grounded in digital empathy theory and prior empirical work (38). Key distinctions were clarified. A subset of 50 comments was coded independently, and Krippendorff’s Alpha reached 0.78. Divergences were resolved through consensus to finalize the coding framework.

## 4 Results

Following the incident, multiple related hashtags quickly converged on Weibo, forming a media-dominated arena of public opinion. The discussions were primarily centered on core issues such as medical decision-making, cyberbullying, and official responses, showing characteristics of concentrated information release, a tightly paced rhythm of dissemination, and broad public participation. Through agenda construction and narrative framing, media outlets effectively directed public attention and emotional resonance, generating a convergent effect around news-driven agendas.

### 4.1 Thematic analysis of Weibo posts

In this study, the analytical corpus was divided into two parts: Weibo posts and comments. It is important to clarify that the term “posts” does not refer exclusively to content published by media outlets. Rather, it mainly consists of topic-related posts initiated by official media accounts and news organizations, while also including a smaller proportion of original posts from ordinary users. However, the latter generally attracted limited engagement and were often overshadowed by large-scale media reporting and reposting. This division aligns with recent empirical findings on Weibo discourse. Xu and Yang (42) identified a “discourse stratification” pattern where posts dominated by media and high-influence accounts function as the core of agenda setting and narrative framing. Hu et al. (39) further confirmed that even non-media posts rarely shape core discussion directions due to their low visibility. Accordingly, Weibo posts here are understood as representing the agenda-setting and framing layer of the discourse, with media acting as primary agenda setters. Comments, by contrast, are almost entirely generated by ordinary users and constitute the responsive and participatory layer. They directly reflect public emotional responses and interactional logic, as noted in both studies.

**Figure 1:**
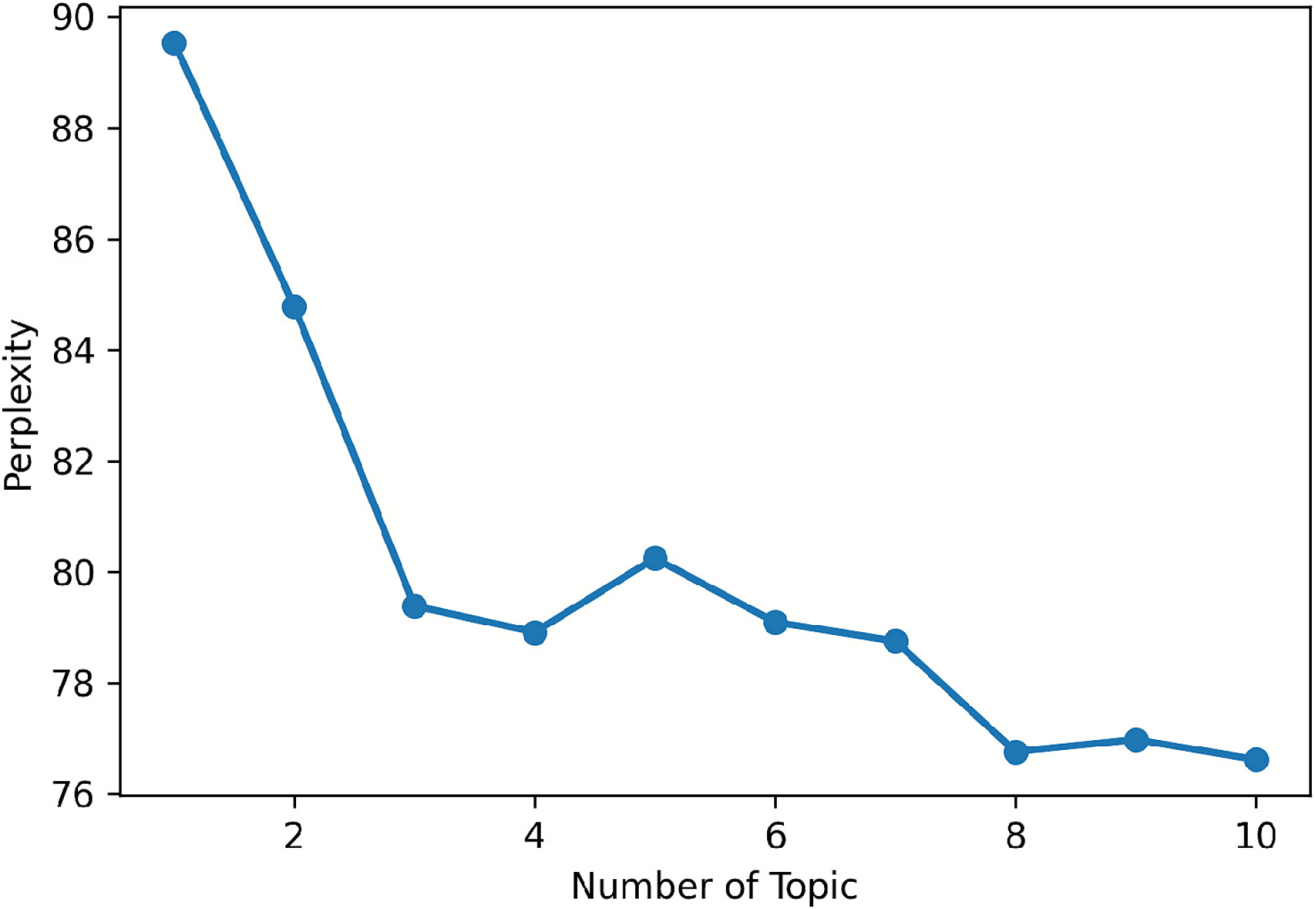
Perplexity curve for post.

**Figure 2:**
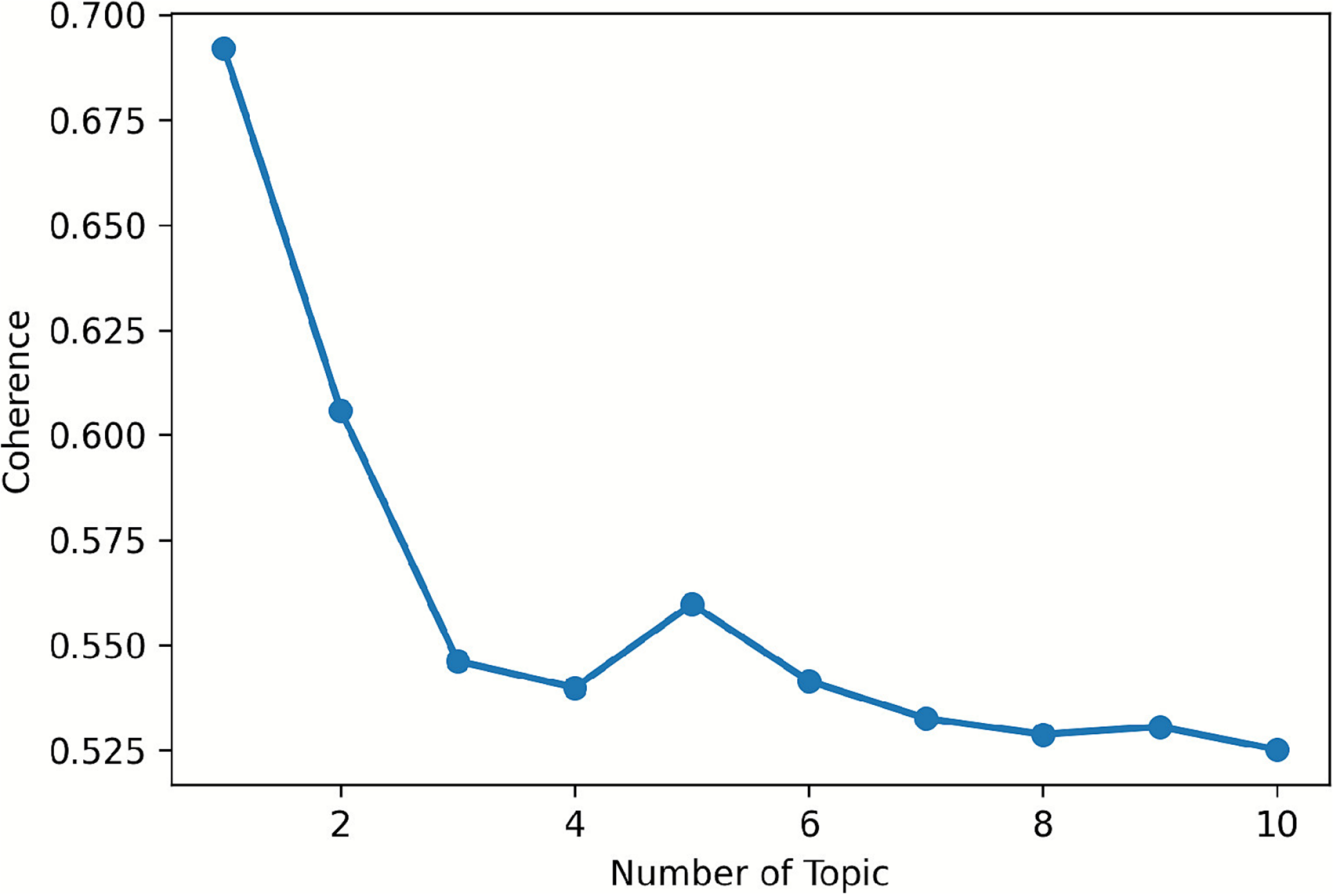
Coherence curve for post.

For the post layer LDA, the study determined the optimal topic number by analyzing perplexity and coherence curves. Perplexity decreased sharply to 4 topics and then stabilized, while coherence stabilized from 4 topics onward (see Figures 1, 2). Thus, 4 topics were selected for the post layer, balancing model accuracy and topic interpretability.

The LDA modeling results of Weibo posts reveal that the online discourse surrounding the Zhoukou Doctor Suicide Incident primarily focused on four major themes: medical treatment disputes, cyberbullying and platform responsibility, media reproduction, and official reports and judicial investigation, as detailed below (Table 1).

**Table 1:** Extracted keywords of main themes.

No.	%	Theme	Keywords
1	16%	Medical decision-making dispute	Mother, uterus, amniotic fluid, embolism, falling to death, saving life, hospital, removal, family
2	6.7%	Cyberbullying and platform responsibility	Cyberbullying, platform, internet, tragedy, violence, responsibility, protection, incident, family
3	7.8%	Media reproduction	Video, reporter, release, hospital, falling to death, building fall, family, obstetrics and gynecology, director”
4	69.4%	Official reports and judicial investigation	Zhoukou, fall, Sixth Hospital, police station, official statement, report, authorities, public voice, husband

*Medical decision-making dispute*. Posts under this theme mainly reconstruct the origin of the incident, focusing on the doctor’s decision to perform an emergency hysterectomy to save the patient suffering from amniotic fluid embolism, a severe obstetric complication. Media reports emphasized the professional rationality and urgency of the medical decision while reflecting ongoing public debates on medical risk, informed consent, and doctor–patient communication. The discourse at this stage was characterized by a mixture of understanding and questioning, forming the initial trigger point of public empathy.

*Cyberbullying and platform responsibility*. This theme is represented by keywords such as “cyberbullying,” “platform,” “internet,” “tragedy,” “violence,” “responsibility,” “protection,” and “family.” It reflects public concern about the doctor’s prolonged exposure to online attacks and criticism of the perceived lack of regulation by social media platforms. Relevant posts and reports discussed online governance and mental health protection, emphasizing the harmful effects of cyberviolence within digital discourse and calling for stronger platform accountability and social responsibility.

*Media reproduction*. Key terms such as “video,” “reporter,” “release,” “hospital,” “suicide,” “fall,” “obstetrics,” and “director” indicate this theme’s focus on the multi-dimensional reconstruction and narrative reproduction of the event by news organizations. The reporting included textual narratives, videos, and short-form clips, showcasing the media’s role in agenda setting and emotional reinforcement. Within this theme, media outlets acted not only as information disseminators but also as emotional framers, shaping both public understanding and affective responses.

*Official reports and judicial investigation*. High-frequency words such as “Zhoukou,” “fall,” “Sixth Hospital,” “police station,” “official statement,” “report,” “response,” and “husband” capture the institutional response phase of the discourse. Official announcements and police investigations provided authoritative clarification and legitimacy, guiding public opinion from emotional catharsis toward rational reflection and calls for accountability. This theme underscores the pivotal role of governmental and judicial intervention in regulating online sentiment and restoring public trust during the later stages of opinion evolution.

Taken together, these four themes construct a comprehensive narrative chain spanning the progression from incident origin to online debate, media representation, and institutional response. The structure highlights the dual function of media discourse in public health crises—informational guidance and emotional regulation—and demonstrates how layered agenda setting contributed to the development of digital empathy within the public sphere.

A one-way ANOVA revealed significant differences among the four themes in terms of reposts, comments, and likes (Table 2). The results show that the theme “Media Reproduction” achieved significantly higher averages in reposts, comments, and likes compared with other themes, indicating that media-related content possessed the greatest visibility and diffusion capacity within the public opinion field. This suggests that the audience paid the most attention to news reports and journalistic narratives.

**Table 2:** Differences in retweets, comments, and likes across themes.

Theme	Retweets	Comments	Likes
Medical decision-making dispute	65.37	39.72	734.49
Cyberbullying and platform responsibility	34.6	25.83	292.08
Media reproduction	149.75	101.09	2098.14
Official reports and judicial investigation	6.46	5.59	80.18
*F*-value	7.792	10.096	10.538
*p*	0.000	0.000	0.000

The second-highest level of engagement appeared in the “Medical Decision-Making Disputes” theme, reflecting the public’s heightened sensitivity to the core medical decisions and the ethical reasoning behind them. The “Cyberbullying and Platform Responsibility” theme ranked next in interaction volume, implying that netizens actively participated in reflecting on platform accountability and the broader online environment. In contrast, the “Official Reports and Judicial Investigation” theme, although rich in authoritative information, generated relatively lower engagement, indicating that such content mainly served explanatory and concluding functions rather than driving emotional participation or sustained attention.

**Figure 3:**
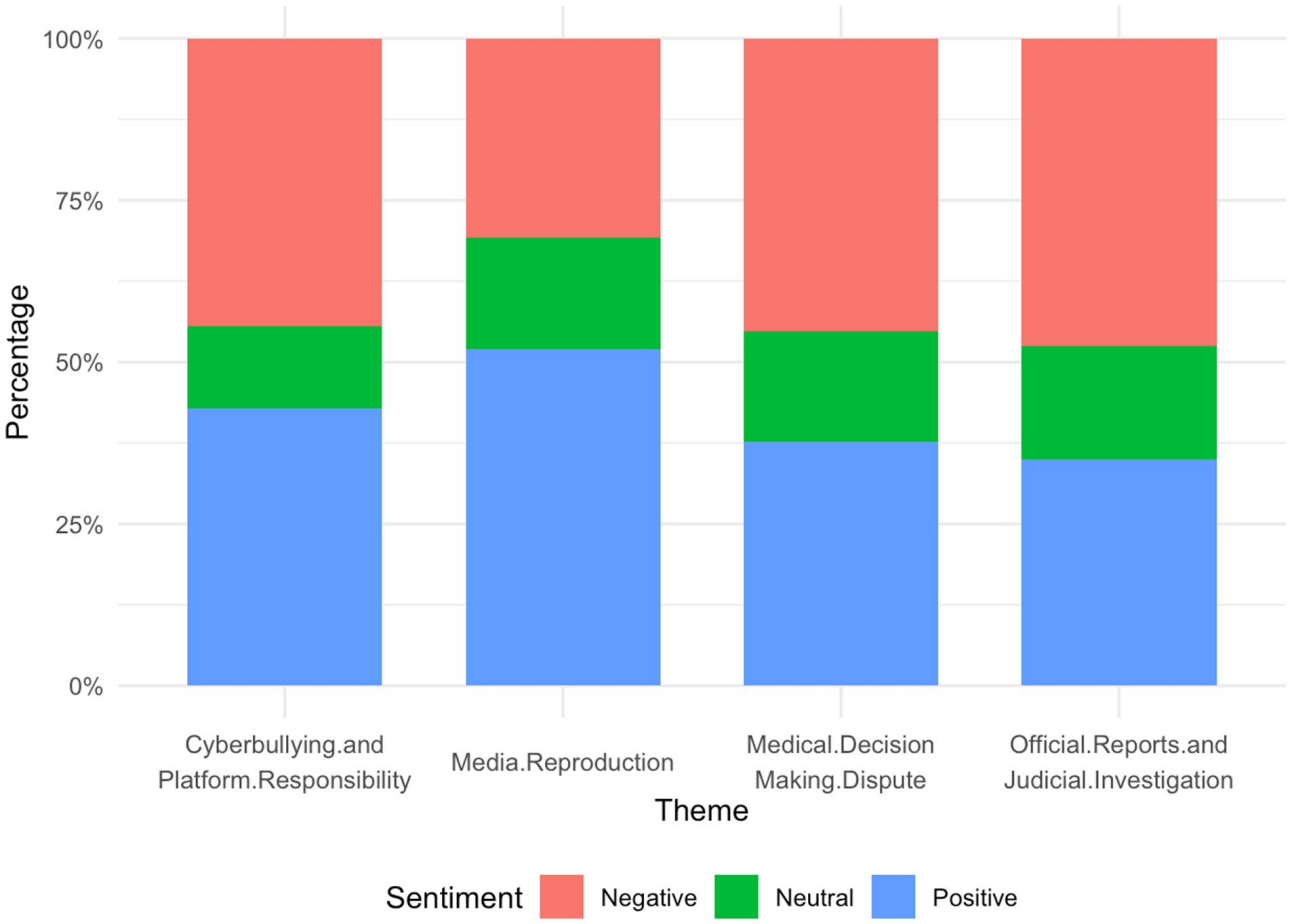
Emotional distribution across different themes.

The four themes exhibited significant differences in emotional distribution which supported H1 (*χ*^2^ = 21.157, *p* = 0.002) (Table 3 and Figure 3). In the themes “Medical Decision-Making Disputes” and “Cyberbullying and Platform Responsibility,” negative emotions accounted for the largest proportion, primarily reflecting anger toward the tragedy, criticism of perceived injustice, and sympathy for the individuals involved. In contrast, the “Media Reproduction” theme contained a higher proportion of positive emotions, as users tended to acknowledge and support the media’s reporting efforts, with some comments expressing rational analysis and fact-based reflection. The “Official Reports and Judicial Investigation” theme showed an overall neutral emotional tendency, suggesting that when encountering authoritative information, the public demonstrated a more fact-oriented and rationally receptive attitude.

**Table 3:** Emotional distribution across different themes.

Theme	Neutral	Positive	Negative
Medical decision-making dispute	52 (17.2%)	114 (37.6%)	137 (45.2%)
Cyberbullying and platform responsibility	16 (12.7%)	54 (42.9%)	56 (44.4%)
Media reproduction	25 (17.1%)	76 (52.1%)	45 (30.8%)
Official reports and judicial investigation	229 (17.6%)	455 (34.9%)	618 (47.5%)
*X* ^2^	21.157		
df	6		
*p*	0.002		

### 4.2 Thematic analysis of comments

**Figure 4:**
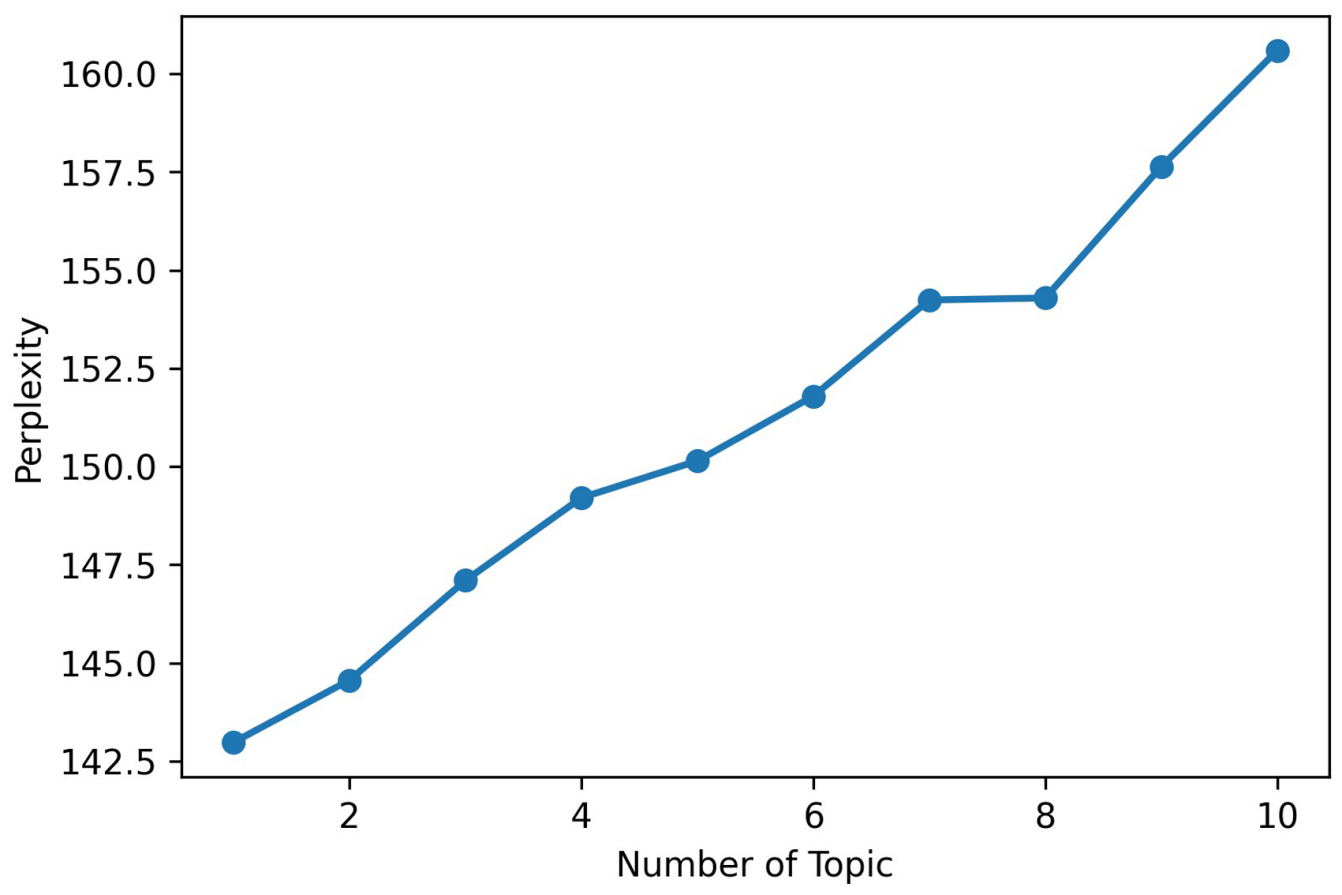
Perplexity curve for comment.

**Figure 5:**
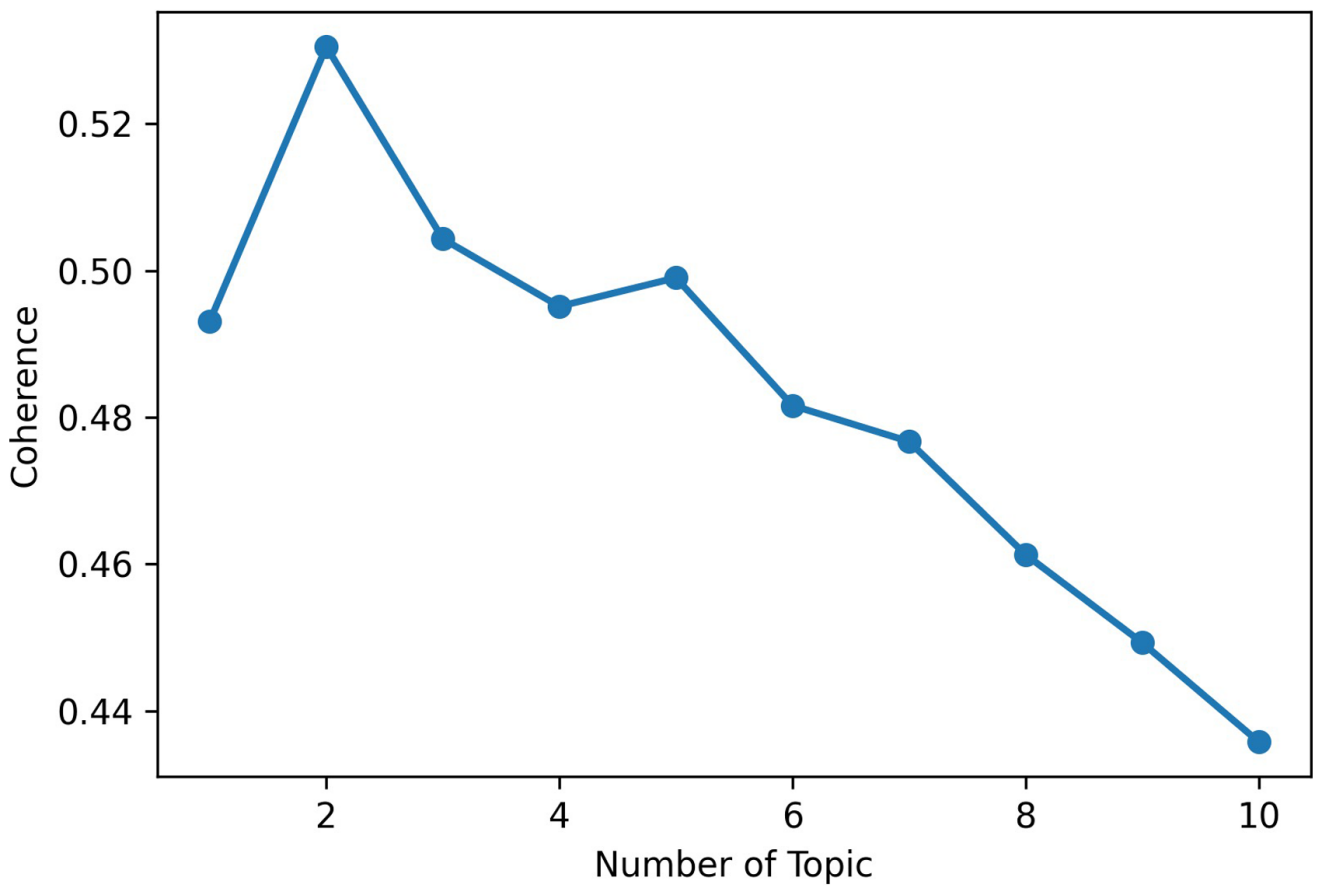
Coherence curve for comment.

For the comment layer LDA, perplexity increased with more topics, and coherence peaked at 2 topics then gradually decreased (see Figures 4, 5). To balance granularity and semantic consistency, 5 topics were chosen, as coherence remained reasonably high at this number, enabling detailed theme exploration.

Based on LDA topic modeling of Weibo comment texts related to the “Zhoukou Doctor Suicide” incident, five core themes were identified, reflecting the multidimensional concerns of the public from medical decision-making to collective online actions (Table 4). These themes reveal the primary directions of public participation in the discourse, encompassing both understanding and reflection on medical practices as well as critique and engagement with institutional accountability, cultural structures, and the online environment.

**Table 4:** Keyword extraction results of comment themes.

No.	%	Theme	Keywords
1	13.3%	Medical decision-making and family conflict	Mother, uterus, amniotic fluid, embolism, family, pregnant woman, father-in-law, infertility, pleading
2	15.1%	Institutional accountability	Should, video, report to police, platform, police, society, severe punishment, responsibility, cyberbully
3	13%	Gender culture	Sadness emoji, child, daughter, son, bride price, son preference, trending, cry, heartbroken
4	8%	Cyber violence and public criticism	Cyber violence, internet, pray, trending, account, news, attention, boost up, terrible
5	50.5%	Physician’s professional dilemma	Doctor, hospital, amniotic fluid, embolism, patient, medical dispute, life, protection, pity

Based on digital empathy theory, this study categorizes the public’s emotional projection across different themes into four types: professional empathy, institutional empathy, cultural empathy, and action-oriented empathy. This classification draws on Unay-Gailhard et al.’s (38) research, which found that empathy on social media is rooted in discussion themes. Our four empathy types align with this “theme-empathy” connection, each tied to distinct contexts—like resonance with professional fields or demands for systemic accountability (Table 5).

**Table 5:** Empathy types derived from comment themes.

Empathy types	Theme	Description
Professional empathy	Medical decision-making and family conflict	The public focuses on medical actions and ethical choices, demonstrating understanding of the dilemmas faced by doctors and patients in life-threatening situations, reflecting emotional identification with professional decisions.
	Physician’s professional dilemma	The public pays attention to the occupational risks and psychological pressures faced by doctors, expressing sympathy for individual tragedies and support for the medical profession.
Institutional empathy	Institutional accountability	The public directs emotional appeals toward institutional actors, emphasizing the responsibilities of the judiciary, authorities, and platforms, reflecting expectations for social justice and institutional protection.
Cultural empathy	Gender culture	The public situates the event within gender structures and cultural contexts, projecting emotions around value conflicts such as “son preference,” reflecting emotional identification with gender equality.
Action-oriented empathy	Cyber violence and public criticism	Based on emotional resonance, the public participates in online actions such as liking, commenting, and reposting to increase topic visibility through algorithmic logic, transforming empathy into strategic emotional expression and participatory dissemination.

*Professional Empathy* originates from individuals’ understanding and identification with medical professionals, and it represents the most prevalent form of empathy expressed in medical-related public discourse (1). Within the themes of “Medical Decision-Making and Family Conflicts” and “Doctors’ Professional Dilemmas,” the public demonstrates compassion toward the tragic outcome by recognizing the ethical dilemmas and risk burdens faced by doctors. This empathy reflects trust and support for professional judgment and exhibits strong cognitive empathy characteristics, as individuals engage in perspective-taking to understand others’ situations and form emotional connections.

*Institutional Empathy* manifests when emotional appeals are elevated to the level of systems and regulations, focusing on the roles of the judiciary, platforms, and governance structures (40). In the “Institutional Accountability” theme, comments frequently call for “thorough investigation” and “severe punishment of cyberbullies,” reflecting expectations for social justice and responsibility attribution. This form of empathy is rooted in moral alignment, emphasizing the compensatory function of institutions, and represents a socially oriented response combining emotional concern with rational demands.

*Cultural Empathy* is grounded in shared socio-cultural contexts, whereby individuals reinterpret incidents through cultural symbols and value identification (8). In the “Gender Culture” theme, discussions frequently mention issues such as “son preference” and “family pressure,” situating the individual tragedy within broader gender structures and cultural contexts. This empathy reveals critical reflections on traditional gender norms and an endorsement of egalitarian values. It is characterized by value-oriented emotional participation, intertwining affective responses with socio-cultural cognition.

*Action-Oriented Empathy* represents a unique form of emotional externalization in digital public spheres (10). It refers to the transformation of emotional resonance into observable participatory actions—such as liking, commenting, and sharing—that enhance the visibility of an issue. Unlike purely expressive emotions, action-oriented empathy integrates affective engagement with platform-aware behavioral participation, reflecting both genuine support and a strategic understanding of recommendation algorithms.

Compared with concepts such as behavioral empathy or active empathy, action-oriented empathy emphasizes algorithm-mediated amplification rather than interpersonal helping behaviors. While behavioral or active empathy usually involves offline assistance, perspective-taking, or prosocial acts, action-oriented empathy focuses on boosting visibility, mobilizing attention, and shaping public sentiment through platform-specific participatory mechanisms.

**Figure 6:**
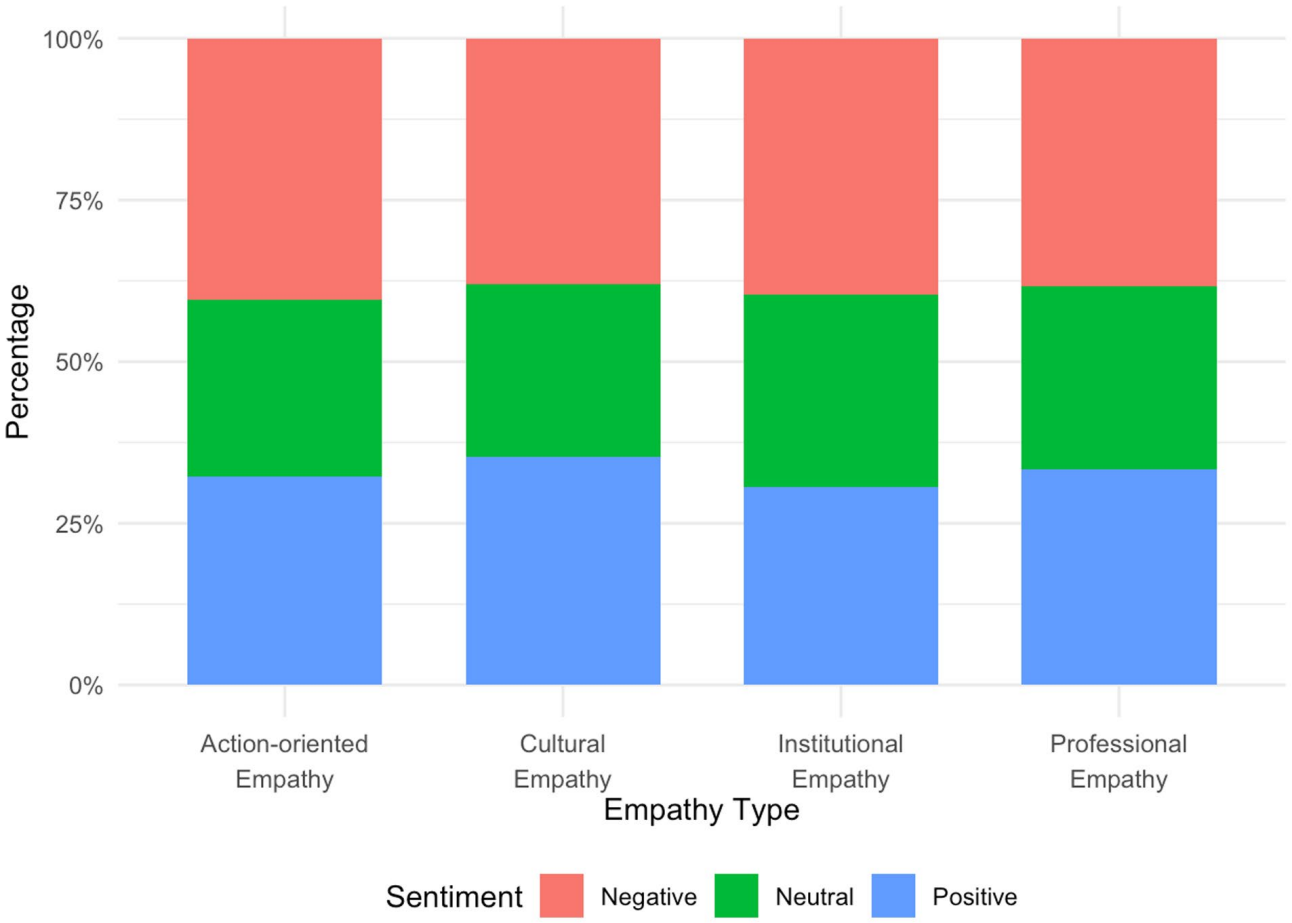
Emotional distribution across different types of digital empathy.

The emotional analysis revealed significant differences in emotion distribution among the four empathy types which supported H2 (*χ*^2^ = 14.414, *p* = 0.025). Overall, negative emotions dominated across all types, indicating that the public’s responses to the incident were primarily shaped by feelings of compassion, grief, and anger. However, the composition of emotional tendencies varied notably across types (see Table 6 and Figure 6).

**Table 6:** Emotional distribution across different types of digital empathy.

Empathy type	Neutral	Positive	Negative
Professional empathy	2,956 (28.2%)	3,504 (33.4%)	4,026 (38.4%)
Institutional empathy	739 (29.8%)	759 (30.6%)	981 (39.6%)
Cultural empathy	573 (26.8%)	754 (35.2%)	814 (38.0%)
Action-oriented empathy	361 (27.3%)	426 (32.2%)	535 (40.5%)
*X* ^2^	14.414		
df	6		
*p*	0.025		

For professional empathy, which had the largest volume, negative emotions accounted for 38.4%, followed by positive emotions (33.4%) and neutral emotions (28.2%). This pattern suggests that while users expressed understanding and support for the doctor’s professional decision-making, their empathy was also accompanied by sorrow over the tragic outcome and anxiety about systemic medical risks—reflecting a complex and ambivalent emotional structure.

In institutional empathy, negative emotions (39.5%) were also dominant, reflecting public dissatisfaction with institutional flaws and criticism of regulatory failures as users called for accountability and systemic reflection.

For cultural empathy, the proportions of positive (35.1%) and negative (37.8%) emotions were relatively close, revealing a polarized and contentious emotional profile. On one hand, some users engaged in constructive reflection by criticizing gender bias and advocating equality; on the other, emotionally charged debates and confrontations between gender standpoints intensified emotional volatility.

Action-oriented empathy exhibited the highest proportion of negative emotions (39.7%), yet also a considerable share of positive emotions (31.7%). This indicates that users discussing cyberviolence and public criticism tended to express stronger and more explicit emotions, often transforming their feelings into visible actions such as “boosting” the post or “making it seen,” demonstrating a perceptual awareness of algorithms and a tendency toward actionable empathy in digital spaces.

The four types of empathy exhibited significant gender differences which support H3 (*χ*^2^ = 13.472, *p* = 0.004) (Table 7). Female users showed consistently higher participation across all empathy types. They demonstrated higher levels of engagement in cultural empathy (65.4%), reflecting their focus on the social and emotional dimensions of the crisis, such as the doctor’s emotional suffering and the cultural implications of the incident. Women were more likely to express concern for vulnerable groups and advocate for emotional support, often framing the issue in terms of broader societal impacts.

**Table 7:** Gender across different types of digital empathy.

Empathy type	Male	Female
Professional empathy	3,993 (38.1%)	6,493 (61.9%)
Institutional empathy	962 (38.8%)	1,517 (61.2%)
Cultural empathy	740 (34.6%)	1,401 (65.4%)
Action-oriented empathy	528 (39.9%)	794 (60.1%)
*X* ^2^	13.472	
df	3	
*p*	0.004	

In contrast, male users showed the highest engagement in action-oriented empathy (39.9%). This form of empathy, based on emotional resonance, motivates individuals to participate in online actions such as liking, commenting, and reposting to amplify the visibility of the topic through algorithmic logic. Male users were more likely to call for increased engagement in the form of comments and reposts, aiming to raise the event’s profile and spread awareness. This type of participation not only facilitates emotional expression but also serves to amplify the issue, particularly in discussions related to combating online violence and ensuring institutional accountability.

#### 4.3 Temporal analysis

**Figure 7:**
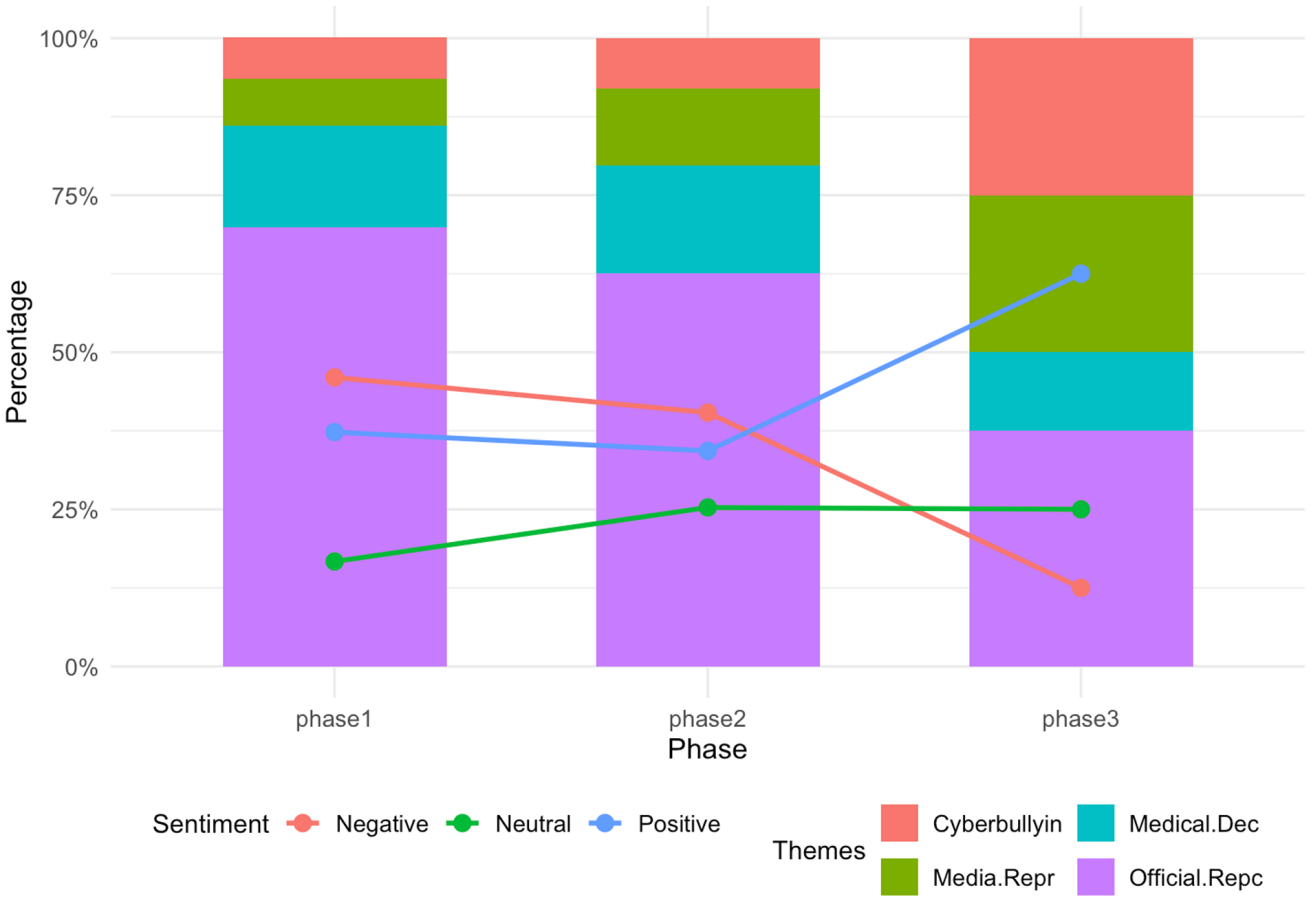
Temporal variation in themes and sentiment across three phases.

**Figure 8:**
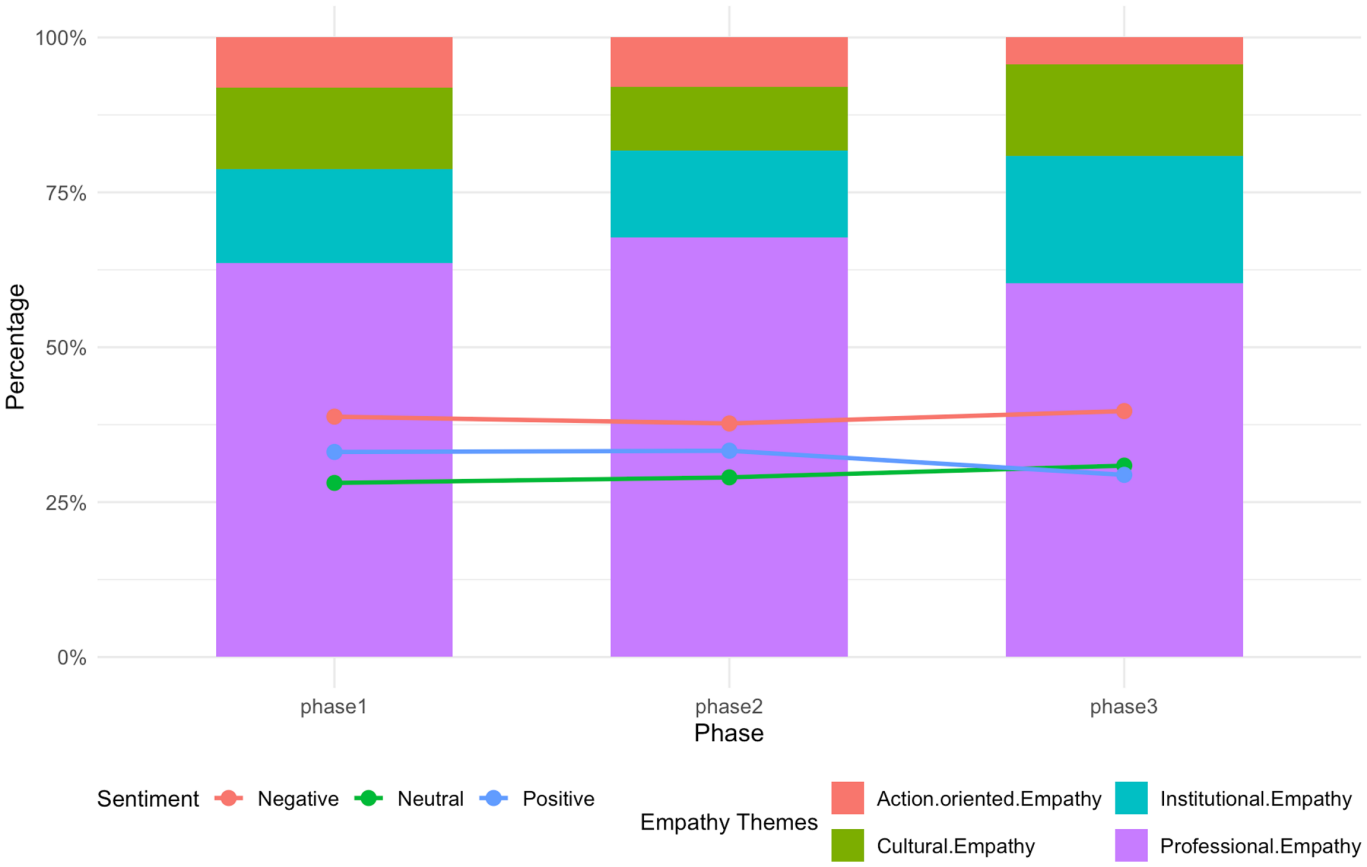
Temporal variation in empathy types and sentiment across three phases.

To explore whether public responses changed as the incident unfolded, the data were divided into three stages based on major shifts in information release and discussion rhythm (41). Stage 1 (Aug 5–10), following the initial official statement and peak media coverage; Stage 2 (Aug 11–20), the period of sustained public debate; and Stage 3 (Aug 21–31), the later phase characterized by declining attention. Although differences did not reach statistical significance, the temporal patterns reveal several meaningful trends across themes, emotions, and empathy types (see Figures 7, 8 for detailed distributions).

*Stage 1*. Public attention in the first stage was driven by the official response and concentrated media amplification. Posts overwhelmingly focused on Official Reports and Judicial Investigation (69.9%), while other themes remained secondary. Negative sentiment was highest (46.0%), reflecting widespread shock, anger, and moral indignation at the doctor’s death. In the comment layer, professional empathy dominated (63.6%), as users expressed support for the doctor’s medical decisions and professional vulnerability. Institutional and cultural empathy were comparatively low at this early stage, indicating that users were still reacting emotionally rather than engaging in moral reasoning or systemic critique. This phase represents the affective flashpoint of the incident.

*Stage 2*. In Stage 2, the thematic landscape diversified. Discussions about Medical Decision-Making Disputes and Cyberbullying and Platform Responsibility expanded, signaling a shift from emotional outburst to interpretive and meaning-making discourse. Negative emotions declined modestly, while neutral and positive expressions increased, suggesting more reflective engagement as users processed additional information. Empathy patterns became more stable: professional empathy remained dominant (67.7%), but institutional empathy and cultural empathy gained presence. This indicates that users were moving from emotional resonance to evaluations of responsibility, institutional norms, and cultural expectations surrounding medical practice and online behavior.

*Stage 3*. Although the number of posts and comments declined in Stage 3, two interesting patterns emerged. First, Cyberbullying and Platform Responsibility and Media Reproduction surged to 25% each, showing that discussions shifted toward structural concerns about platform governance, media ethics, and systemic accountability. Second, positive emotions increased markedly (62.5%), pointing to processes of closure, reframing, or forward-looking reflection. Empathy patterns changed as well. Institutional empathy rose (20.6%), reflecting greater concern for systemic protection of healthcare workers, while action-oriented empathy decreased (4.4%), suggesting reduced mobilization (“push it to the top”) as public attention subsided and users shifted toward normative reflection rather than active amplification.

## 5 Discussion

This study examines Weibo discussions surrounding the “Zhoukou Doctor’s Suicide” incident and identifies how public opinion and digital empathy unfold in a public health crisis. The findings show a dual dynamic in which media-driven agenda setting shapes the early structure of public discourse, while public emotional resonance expands and deepens it. Rather than remaining at the level of information exchange, online participation evolves into multi-layered empathy expressions that integrate cognitive, emotional, and action-oriented dimensions.

Analysis of Weibo posts identified four dominant themes: medical decision-making disputes, cyberbullying and platform responsibility, media reproduction, and official reports and judicial investigation. These themes reflect a chain-like evolution of public opinion from incident origins and online debates to media representation and institutional response. Media coverage, particularly reports highlighting the doctor’s ethical decision and professional risks, amplified public empathy and set the emotional tone of early discussions. As attention shifted toward cyberbullying and institutional accountability, public emotions diversified into grief, anger, and reflection, demonstrating a transition from emotional catharsis to collective introspection. This pattern indicates that media reports functioned not only as channels of information dissemination but also as emotional triggers shaping the focal points and trajectories of public empathy.

At the comment level, four distinct empathy types emerged: professional empathy, institutional empathy, cultural empathy, and action-oriented empathy. Professional empathy centered on understanding the doctor’s professional challenges and ethical dilemmas, accompanied by feelings of compassion, regret, and anxiety. Institutional empathy focused on the expectation of accountability and systemic justice, reflecting public calls for platform regulation and legal intervention. Cultural empathy revolved around gender issues and traditional norms, revealing a critical reflection on patriarchal expectations and family structures. Action-oriented empathy manifested as the translation of emotions into visible actions through comments, likes, reposts, and phrases such as “boost it to the top,” illustrating users’ algorithmic perception and emotional motivation to influence platform visibility. Sentiment analysis showed significant emotional differences across empathy types (*χ*^2^ = 14.414, *p* = 0.025), with negative emotions being dominant but coexisting with substantial positive and neutral expressions. Female users exhibited higher levels of engagement across all empathy types, suggesting stronger emotional sensitivity and participatory motivation.

The temporal patterns observed in this study demonstrate that digital empathy in public health crises unfolds through a dynamic, staged, and multi-layered process. In the early outbreak stage, media reporting and official statements create a moment of affective shock, activating rapid emotional contagion and producing high levels of negative sentiment. As discussions progress, thematic differentiation emerges and users begin to engage in interpretive work, shifting from emotional release to debates about medical ethics, cyberviolence, and institutional accountability. In the later phase, as attention stabilizes and more information becomes available, public expressions increasingly reflect normative reflection, with empathy directed toward broader structural concerns and expectations for systemic reform.

These dynamics suggest that digital empathy is not a static emotional response but a relational and socio-technical practice shaped jointly by media narratives, issue characteristics, and platform mechanisms. Emotions are continually reframed as users interact, reinterpret events, and adjust their empathic stance in response to evolving information environments. The transition from emotional arousal to reflective evaluation illustrates how empathy functions simultaneously as a psychological experience, a meaning-making process, and a form of digitally mediated participation. In this sense, digital empathy becomes a key mechanism through which publics negotiate moral positions, mobilize collective attention, and articulate expectations for institutional action in algorithmically structured communication spaces.

Practically, the findings highlight the need for multi-stakeholder collaboration to foster rational and constructive empathy in digital spaces. Media organizations should strengthen empathic sensitivity in crisis reporting to avoid emotional manipulation or moral polarization. Platforms must strengthen algorithmic governance, increase the detection of harmful content, and respond more quickly to posts that involve online violence. They should improve reporting tools, raise the sensitivity of content filters, and intervene earlier when large-scale attacks begin to form. Government agencies and institutions should provide transparent communication and psychological support to address public emotional demands. Citizens, in turn, should cultivate reflective expression and responsible empathy, promoting a healthier and more dialogic online environment, and approach public health events rationally without provoking unnecessary group conflicts.

Although Weibo provides valuable insights into public discussions on health-related and social issues, the use of social media data inevitably involves potential biases. User-generated content tends to reflect the behaviors and opinions of more active, digitally literate, and urban populations, which may not be representative of the general Chinese population. Moreover, algorithmic recommendation systems and platform-specific affordances can amplify certain topics or emotional expressions, influencing the visibility and circulation of posts. These factors may introduce selection and self-presentation biases that should be taken into account when interpreting the findings. Future research could combine data from multiple platforms or employ survey-based validation to improve representativeness and robustness.

From an ethical perspective, this study relied solely on publicly available Weibo data and did not involve any identifiable personal information or private communication. Nevertheless, research involving online harassment and emotional expression requires careful consideration of potential psychological harm. While the data are open to the public, reanalyzing or citing sensitive content may inadvertently evoke emotional distress for individuals or groups affected by such incidents. Therefore, this study treated all materials with caution and aimed to promote understanding and empathy rather than reproduce or amplify harm. Future research should continue to balance analytical rigor with ethical sensitivity when working with emotionally charged or vulnerable online communities.

In conclusion, digital empathy in public health crises represents an integrated process linking emotion, cognition, and social action. Through the case analysis of the Zhoukou doctor incident, this study reveals how empathy is generated, expressed, and transformed within digital public opinion, offering theoretical contributions to the study of affective mechanisms and practical implications for empathetic governance and crisis communication in the digital era.

## Data Availability

The raw data supporting the conclusions of this article will be made available by the authors, without undue reservation.
